# Robot-Assisted Radical Cystectomy (RARC) and Intracorporeal Studer Neobladder: Surgical Technique, Feasibility, and Early Functional and Oncological Outcomes

**DOI:** 10.7759/cureus.90294

**Published:** 2025-08-17

**Authors:** Omar Elsandoby, Mohammad Masoud, Danny Darlington, Misra Siddarth, Zainab Motiwala, Tsung Chang, Hanif Motiwala, Matteo Massanova

**Affiliations:** 1 Urology, Royal Surrey County Hospital, Guildford, GBR; 2 Urology, Southend University Hospital, Southend, GBR; 3 Uro-Oncology, Max Super Speciality Hospital, New Delhi, IND; 4 Urology, Armed Forces Medical College, Pune, IND; 5 Faculty of Medicine, Jawaharlal Nehru Medical College (JNMC) Aligarh Muslim University, Aligarh, IND

**Keywords:** functional outcome, icud, neobladder, rarc, studer pouch

## Abstract

Introduction: Radical cystectomy can be associated with a high risk of perioperative morbidity and an impact on urinary continence and sexual function. Different techniques of intracorporeal urinary diversion (ICUD) have been described with the aim of improving the postoperative functional outcome. We will talk about our experience in robot-assisted radical cystectomy (RARC) and ICUD.

Methodology: This is a retrospective study including patients who underwent RARC and intracorporeal Studer neobladder. The oncological outcomes were assessed during the follow-up with a CT CAP that was done at 6, 12, and 24 months. Urinary continence was assessed at 12 months postoperatively. The number of pads was recorded separately for the daytime and nighttime. In case of hypercontinence, frequency of clean intermittent self-catheterization was recorded as well.

Results: A total of 12 patients were included in our study. Our data showed that neobladder configuration resulted in a longer operative time, but this was not associated with a higher complication rate or length of hospital stay. Most of the patients (91%) were continent during the day and used 0-1 pad only, also a large proportion of the patients (63.6%) achieved good nighttime continence and used 0-1 pad only during the night. Daytime hypercontinence was reported in 45.4% of patients and nighttime hypercontinence was reported in 27.2% of patients.

Conclusion: While RARC and neobladder are complex procedures that require specialized surgical expertise, they are technically feasible, safe and can offer hope for patients with bladder cancer to have a more natural and functional alternative to the traditional ileal conduit.

## Introduction

Bladder cancer is the 11th most common cancer in the UK, accounting for about 3% of the newly diagnosed cancer cases. It is the seventh commonest cancer in men and the 17th commonest cancer in women in the UK. Around 10,500 new cases are diagnosed on a yearly basis. Bladder cancer is the ninth most common cause of cancer-specific mortality in the UK, accounting for 3% of all cancer deaths. Around 5,600 deaths occur due to bladder cancer every year. The 10-year disease-specific survival of bladder cancer is estimated to be around 46.3%; this tends to be higher in the cases diagnosed before the age of 55 (57.4%) compared to the cases diagnosed after the age of 75 (31.5%) [1].

NICE guidelines recommended either radical cystectomy or radiotherapy with a radiosensitizer to people with muscle‑invasive urothelial bladder cancer. They also recommend with radical cystectomy a urinary stoma, or alternatively a continent urinary diversion (neobladder or a catheterisable reservoir) if there are no strong contraindications such as cognitive impairment, impaired renal function, or significant bowel disease [2].

Not only the oncological result, but also the functional outcome is of profound importance, especially in young patients. Radical cystectomy can be associated with a high incidence of perioperative morbidity, including an impact on urinary continence and sexual function [3].

Different techniques have been described with the aim of improving the postoperative functional outcome [4]. Robot-assisted radical cystectomy (RARC) and intracorporeal urinary diversion (ICUD) were associated with a better functional outcome in terms of urinary continence, while erectile function preservation depends on other factors like nerve preservation, age, and comorbidities [5].

## Materials and methods

Patient population

Data was collected for patients who underwent RARC with intracorporeal orthotopic neobladder done by a single experienced surgeon in Southend University Hospital, UK, in the period from January 2020 to January 2024.

Surgical technique

**Figure 1:**
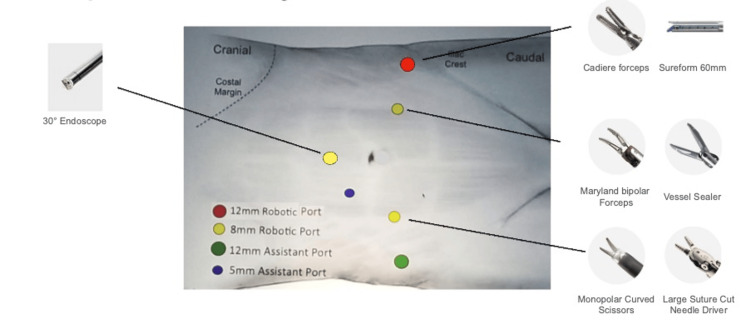
Trocar placement for RARC © Matteo Massanova, Southend University Hospital, UK RARC: Robot-assisted radical cystectomy

The patient is placed in a steep Trendelenburg position (25-30 degrees). A urethral catheter is placed in a sterile manner after draping. In female patients, a vaginal swab is placed to help identify the vaginal vault intraoperatively. We use the da Vinci X or Xi robot from Intuitive Surgical (Sunnyvale, United States). The trocar configuration is shown in Figure 1.

**Figure 2:**
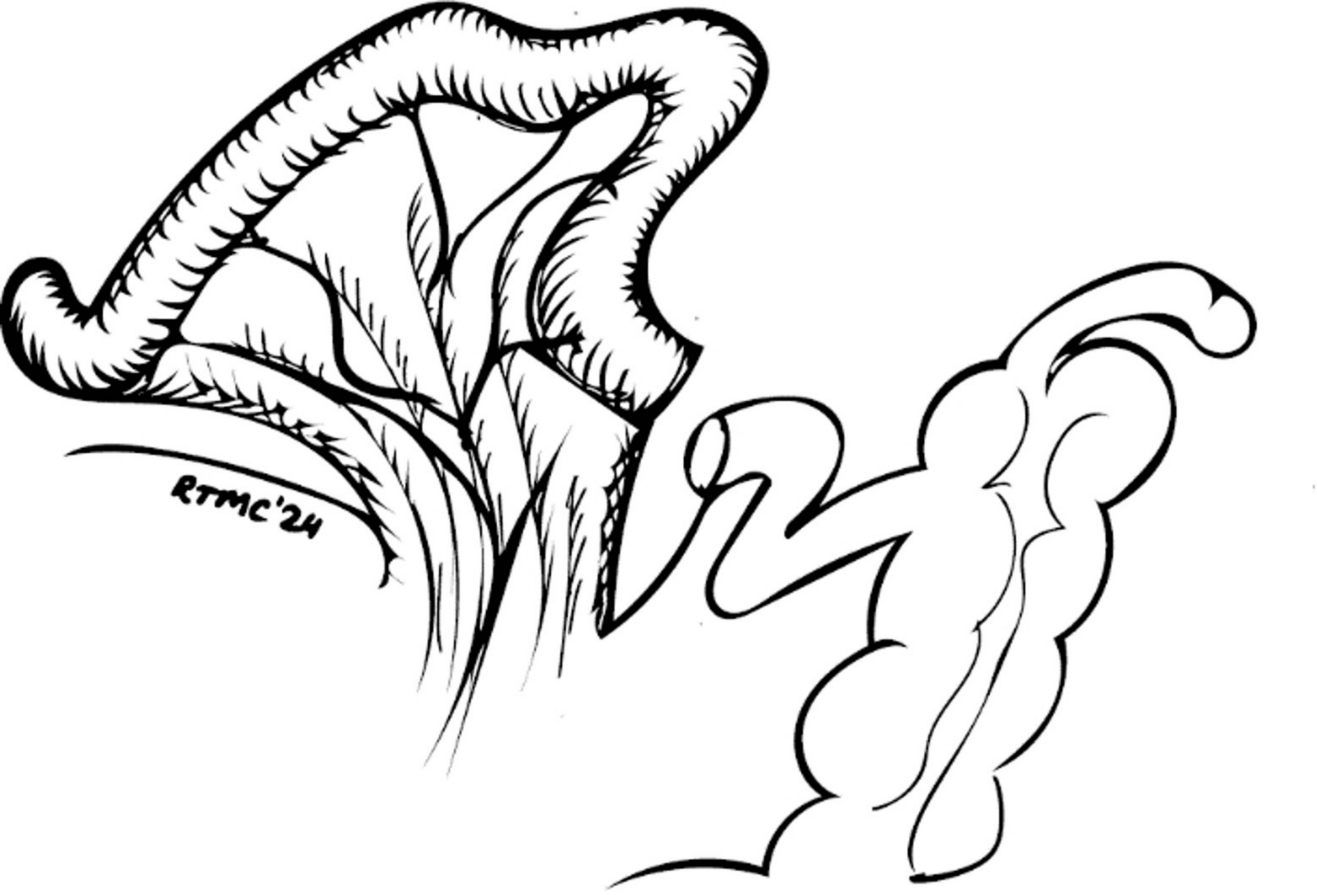
The isolated ileal loop © Tsung Chang, Southend University Hospital, UK

The pneumoperitoneum is adjusted to 8-10 mmHg. The ureters are mobilized on both sides down to the urinary bladder. The posterior plane is developed. In male patients, the peritoneal reflection on the recto-vesical pouch is incised, and the plane is developed down to the apex of the prostate. In female patients, an incision is made on the posterior vaginal fornix, which is identified with the aid of a vaginal swab previously positioned. The lateral plane is developed lateral to the medial umbilical ligaments down to the endopelvic fascia. The ureters and the medial umbilical ligaments are clipped as low as possible. The lateral pedicles are controlled with da Vinci Vessel Sealer®. The urachus is incised, and the Retzius space is developed. The bladder is dropped down, and the urethra is identified and dissected circumferentially. To prevent urine spillage, a Haem-o-lock® is applied to close the urethra before this is divided. The dorsal vein complex is sutured with 3/0 V-Loc™ to obtain haemostasis. A bilateral extended pelvic lymphadenectomy is done. In female patients, the specimen is removed transvaginally, and the vagina is then reconstructed with 3/0 V-Loc™. In male patients, the specimen is removed at the end through a supraumbilical incision. An intracorporeal modified Studer pouch is then constructed. A 45 cm segment of the distal ileum is taken 20 cm proximal to the ileocecal junction (Figure 2).

**Figure 3:**
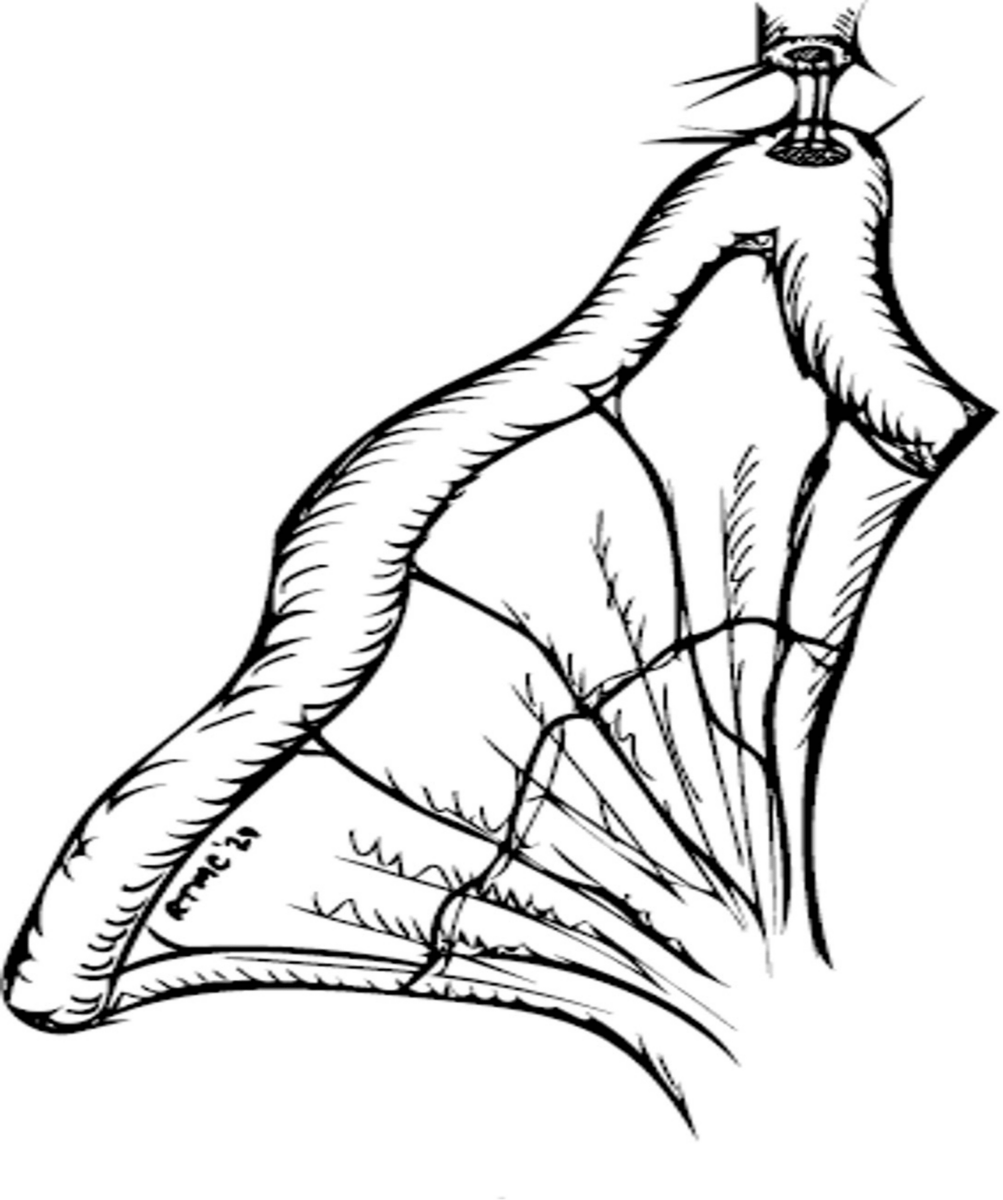
The urethro-ileal anastomosis © Tsung Chang, Southend University Hospital, UK

The bowel continuity is restored with da Vinci SureForm 60™. The urethro-ileal anastomosis is done first after taking a stabilizing stitch between the Denonvilliers' fascia and the ileal loop. The anastomosis is done using double-armed 2/0 Quill™ 9 cm from the distal end of the ileal loop (Figure 3).

**Figure 4:**
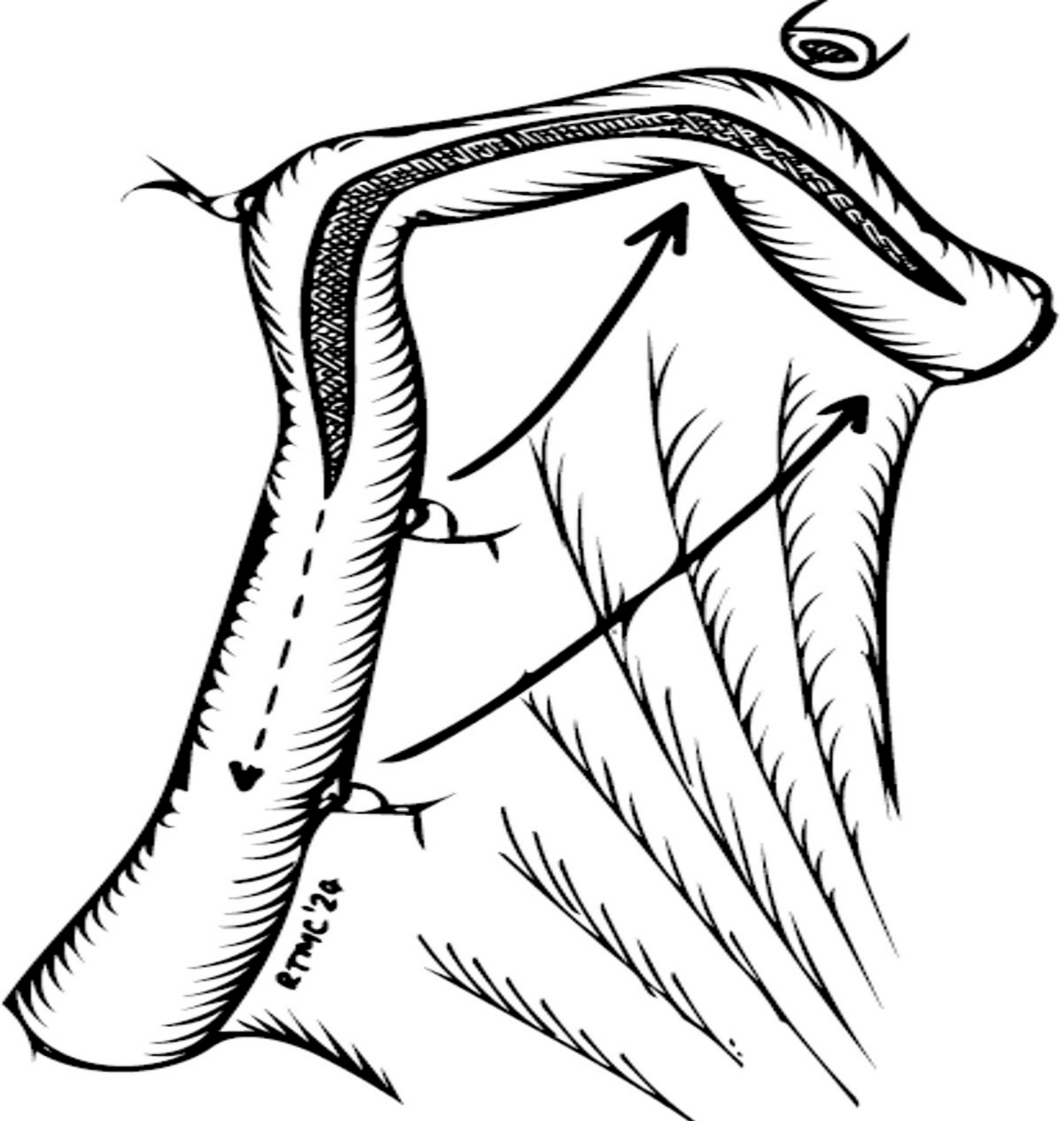
Detublurization, placement of the marker stitches and configuration of the first fold © Tsung Chang, Southend University Hospital, UK

The ileal loop is then detubularized sparing the proximal 9 cm, which will act as a chimney. The ileal loop is marked with marker stitches every 9 cm; the distal one is placed on the outer side, and the proximal two are placed on the inner side for easier handling. The first folding is then performed by moving the proximal marker stitch to the distal end, and the second one is moved under the anastomosis (Figure 4).

**Figure 5:**
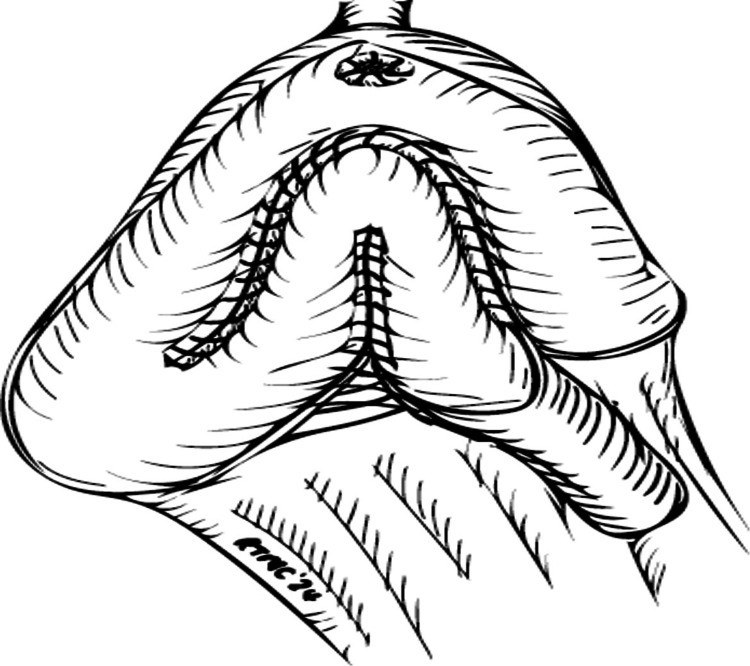
Suturing of the posterior plate © Tsung Chang, Southend University Hospital, UK

The two folds of the posterior plate are then sutured with 3/0 V-Loc™ between the marker stitches (Figure 5).

The distal half of the anterior wall is sutured. The uretero-ileal anastomosis is done using the Wallace technique for benign cases and Bricker for cancer cases, using interrupted 4/0 Monocryl™ with placement of mono J stents. The stents are brought out through the anterior wall of the pouch, then outside through one of the ports. The closure of the anterior wall of the pouch is then completed. A Robinson’s drain is left through the left iliac fossa port for a few days postoperatively. The ERAS protocol is applied whenever possible. Flushing of the neobladder is done starting from day 2 postoperatively, and the patient is taught to do so on discharge. A large-bore urethral catheter is left for four weeks and removed after a cystogram has shown complete healing and no urinary leak. The ureteric stents are pulled out after 10days.

Oncological outcome

The final histology was reviewed for every patient. A CT abdomen and pelvis with contrast was done postoperatively at 6, 12, and 24 months to assess for local recurrence and distant metastasis.

Functional outcome

Urinary continence was assessed at the 12th month postoperatively. The number of pads was recorded separately for the daytime and nighttime. Continence was defined as 0-1 pad, and complete continence was defined as dryness with no need to use pads. In case of hypercontinence, frequency of clean intermittent self-catheterization (CISC) was recorded as well.

Statistical analysis

Descriptive statistical analyses were performed, with the median (range) reported for continuous variables and the frequency (proportion) for categorical variables.

## Results

Patient population

A total of 12 patients were included in our study. Table 1 shows the detailed characteristics of these patients. There were nine male patients (75%) and three female patients (25%). The median age at diagnosis was 62 years (range 34 - 80). The median BMI was 25.64 kg/m^2^ (range 24.5 - 32). All patients had an ASA score of 2. A total of eight patients (66.6%) had muscle-invasive bladder cancer (MIBC), three patients (25%) had a high-risk non-muscle invasive bladder cancer (NMIBC) and one patient (8.3%) had refractory overactive bladder symptoms. Neoadjuvant chemotherapy (NAC) was administered in six of the MIBC patients. Preoperative transurethral resection of the prostate (TURP) was done in six of the male patients. All patients had RARC and an intracorporeal Studer pouch. Extended lymph node dissection was done in all patients except the overactive bladder patient. The median total operative time was 300 min (range 270 - 390). The median amount of estimated blood loss was 100ml (range 50 - 250). There were no reported intraoperative complications. The median length of stay in the hospital was 8.5 days (range 3 - 20).

**Table 1:** Patient characteristics and perioperative data TURP: Transurethral resection of the prostate; EBL: estimated blood loss; LOS: length of stay; NAC: neoadjuvant chemotherapy

Parameter	Result
Age in years median (range)	62 (34-80)
Sex	
Male n (%)	9 (75)
Female n (%)	3 (25)
BMI in kg/m^2^ median (range)	25.64 (24.5-32)
Clinical T stage	
cTa n (%)	1 (8.3)
cT1 n (%)	2 (16.6)
cT2 n (%)	8 (66.6)
NAC n (%)	6 (50)
Preop TURP n (%)	6 (50)
Op time in minutes median (range)	300 (270-390)
EBL in ml median (range)	100 (50-250)
LOS in days median (range)	8.5 (3-20)

Oncological results

This section applies only to 11 patients who had bladder cancer. A total of three patients had pT0 (27.2%), one patient had pTis (9%), one patient had pT1 (9%), three patients had pT2 (27.2%) and three patients had pT3 (27.2%) in the final histology. Of the patients who had pT0, PTis or pT1, 2 had originally MIBC which was down staged after neoadjuvant chemotherapy (NAC). Only one patient (9%) had positive lymph nodes (pN1). A total of four patients had incidental prostate cancer in the final histology (44.4% of the male patients). None of the patients had positive surgical margins. Table 2 shows the detailed oncological results. During the follow-up period, two patients had local recurrence. One of them was pT2 and the other was pT0 in the final histology. Both were pN0 and R0. The neobladder was converted to an ileal conduit in one of them.

**Table 2:** Oncological data

Parameter	Result
Pathological T stage	
pTis n (%)	1 (9)
pT0 n (%)	3 (27.2)
pT1 n (%)	1 (9)
pT2 n (%)	3 (27.2)
pT3 n (%)	3 (27.2)
Pathological N stage	
PN0 n (%)	10 (91)
PN1 n (%)	1 (9)
Surgical Margins	
R0 n (%)	11 (100)
R1 n (%)	0
Concomitant prostate cancer	
Present n (%)	4 (44.4)
Absent n (%)	5 (55.6)
Recurrence	
Yes n (%)	2 (18.1)
No n (%)	9 (81.9)

Functional results

Urinary continence was assessed at the 12th-month follow up. One of the patients had a conversion to ileal conduit after local recurrence, so this section applies only to a total of 11 patients. Continence data are detailed in Table 3. Regarding the daytime continence, a total of five patients (45.4%) were completely continent during the day without the use of any pads, five patients (45.4%) reported the use of one pad during the day, and one patient (9%) reported using two pads during the day. Daytime hypercontinence was reported in five patients (45.4 %) who used CISC to empty their neobladder at variable intervals during the day. Regarding the nighttime continence, a total of four patients (36.3%) were completely continent during the night without the use of any pads, three patients (27.2%) reported the use of one pad during the night, three patients (27.2%) reported using two pads during the night and one patient (9%) was incontinent and used more than three pads during the night. Nighttime hypercontinence was reported in three patients (27.2%) who woke up and used CISC to empty their neobladder during the night.

**Table 3:** Continence data

Parameter	Patients n (%)
Daytime continence	
Zero pad	5 (45.4)
One pad	5 (45.4)
Two pads	1 (9)
Three pads	0
Hypercontinence	5 (45.4)
Nighttime continence	
0 pads	4 (36.3)
1 pad	3 (27.2)
2 pads	3 (27.2)
3 pads	1 (9)
Hypercontinence	3 (27.2)

## Discussion

The intracorporeal Studer ileal neobladder reconstruction was first described in 2011 [6]. This technique results in a low-pressure urinary reservoir with high compliance [7,8]. An important component of this surgical technique is the use of a detubularized bowel segment, which primarily serves two functions: preventing the neobladder contraction and reducing the length of intestine required for the neobladder reconstruction [9,10]. With its standardized and simple technique, it has gained widespread recognition and attention across the globe [11,12]. The majority of the studies that focus on neobladder reconstruction have used the Studer neobladder reconstruction technique [13]. The functional outcomes remained a major concern for patients undergoing radical cystectomy. For that reason, there is a continuous development and modification of the urinary diversion techniques [5]. While ileal conduit urinary diversion is a relatively easier procedure, the creation of orthotopic neobladder is a technically more complex and longer procedure [5]. Our study showed that the neobladder reconstruction resulted in a longer operative time (median of 300 min compared to 210 min), but this was not associated with a higher Clavien-Dindo complication rate or longer median length of hospital stay when compared to the ileal conduit urinary diversion. Similarly, Obara et al. (2006) reported a mean operative time of 382 minutes with a SD of 83 minutes, with a mean blood loss of 1992 ml (SD of 671 ml) [14]. Boonchai et al. (2023) found, in their study, the mean operative time for the Studer technique to be 290 minutes (IQR of 242.5-350 min), which was lower than 300 minutes for the Y pouch technique [15].

Studer neobladder reconstruction following RARC generally offers excellent functional outcomes in terms of continence, voiding function, and sexual function [5]. Most patients achieve a satisfactory or even excellent quality of life after this procedure. When assessing the functional outcome in our cohort after a year of follow-up, it was obvious that most of the patients (91%) were continent during the day and used 0-1 pad only, also a large proportion of the patients (63.6%) achieved a good nighttime continence and used 0-1 pad only during the night. In their comparison between the Y pouch neobladder and Studer technique, Boonchai et al. found that continence recovery was seen in both the techniques with 54.1% of the total patients who underwent the Studer technique achieving nighttime continence and reporting the nighttime pad usage to be 1 ± 1.1 (median ± IQR) at 12 months. On the contrary, 75% of those who underwent the Studer technique achieved daytime continence with a daytime pad usage of 0.7 ± 0.7 (median ± IQR) [15]. Our findings were also supported by the retrospective analysis done by Lantz et al. (2010) who found the 12-month nighttime and daytime continence achievement in 70% and 89% of the patients, respectively [16].

In our cohort, we found 27.2% of the patients reporting hypercontinence, which was determined by the frequency of CISC. It is primarily the inability to void with a residual urine volume of more than 150 ml [17]. Previous studies that have focused on hypercontinence found the level of urethral resection to be an important contributing factor. With a higher level of urethral resection, the hypercontinence rates were reported to be higher [18,19]. Anderson et al. (2012) studied the functional urinary outcomes in 51 patients who underwent orthotopic neobladder reconstruction and found 30.6% of the patients with hypercontinence [17]. On similar lines, Ismail et al. (2008) considered 39 women who underwent orthotopic neobladder reconstruction and contributed to the current literature by noting that hypercontinence was reported in 11 patients (28.2% of the patients) [20]. It is essential to consider that the patient population included in our study was 9 males (75%) and 3 females (25%). This necessitates the need for more studies that focus on hypercontinence outcomes after Studer neobladder reconstruction in patients with a fair representation of both the genders.

Sexual function after RARC with neobladder is variable and depends on factors like pre-existing erectile dysfunction, nerve-sparing techniques, and individual response to surgery [5]. Some studies report preservation of erectile function in a significant proportion of patients following RARC with neobladder [21,22]. However, sexual dysfunction may still occur in some cases, particularly in patients with pre-existing conditions or who undergo extensive surgery [5,23]. Martini et al. (2023) reported the potency without the need for medication and with a PDE-5 inhibitor at 12 months after surgery to be 31% and 24%, respectively [5]. We described potency as the maintenance of an erection enough for sexual intercourse [24]. However, in our cohort, we couldn’t assess the erectile function as most of the patients who had sexual sparing surgery (six patients) did not complete the IIEF questionnaire on time for different reasons, but we will have an assessment of the sexual function on a long-term basis [25].

The oncological outcomes and the complications after the Studer technique must be emphasized because it is being used widely for the surgical removal of bladder cancer [26]. Tyritzis et al. (2013) analyzed the outcomes of the modified Studer neobladder reconstruction in 70 patients, in whom the pathological T stage, including pT1 and above, was seen in 52.85% while the pN0 stage was seen in 80% of the cases. Additionally, prostate cancer detection was seen in 44.8% of the patients, which was similar to our study, where we found 44.4% to have concomitant prostate cancer [27]. 63.6% of our cohort had a pathological stage of pT1 and above, while 91% of the patients had pN0 stage. Our analysis reported a recurrence of cancer in 18% of the patients, which was similar to the recurrence rate of 18.6% that was noticed by Tyritzis et al. [27]. In a case series described by Nazim et al. (2012), 38 patients underwent the Studer neobladder reconstruction, and early complications in the first three months postoperatively were seen in 27% of the patients [28]. Some of the notable renal complications are overall function decline, hydronephrosis, and atrophy [29,30]. Tanaka et al. (2005) analyzed the outcomes of 57 patients with the Studer neobladder and reported complications like urethroileal anastomotic stricture and inguinal hernia in 3.5% and 12.8% of the patients, respectively [31]. Studies have highlighted the importance of adhering to strict bladder emptying protocols to reduce the complications [30,32,33].

Urodynamic studies have been performed on the Studer neobladder, which highlight the favorable long-term outcomes [26]. Kim et al. (2017) explored urodynamic patterns, which can be grouped based on capacity and compliance into ideal parameters, small capacity with low compliance, and large capacity with high compliance. Furthermore, two important factors that were associated with better urodynamic outcomes were found to be younger age and male gender [34]. Porru et al. (1994) studied the urodynamic outcomes in 18 patients with Studer neobladder and found that there is a low filling pressure at high-level filling, with pressure levels usually below 20 cm H2O [35]. A comparison was made between patients with Studer neobladder and the Y pouch neobladder, with an important finding of a post-void residual volume of 46 ml in the Studer group compared to 20 ml in the Y pouch group. The compliance in both types was almost similar, with the majority of the patients having a compliance of 30 ml/cm H2O [15]. Overall, the Studer neobladder has shown satisfactory results in patients above 70 years of age with good continence up to three years postoperatively and appears to show superior functional outcomes compared to the traditional urinary diversion technique like the ileal conduit [14]. The functional and oncological outcomes of the Studer neobladder reconstruction analyzed in various studies are described in Table 4.

**Table 4:** Studies describing Studer neobladder reconstruction

Study Name/Year	Type of Observational Study (Retrospective/Prospective)	Patients with Studer Neobladder (Total Sample Size)	Urinary Functional Outcomes	Renal Function Assessment	Oncological Outcomes	Survival Rates/Recurrence Rates
Martini et al., 2023 [5]	Retrospective	534 (732)	Daytime and nighttime urinary continence outcomes, Erectile function, hypercontinence	None	Pathological T staging	None
Moeen et al., 2016 [9]	Prospective	60	Daytime and nighttime continence status, mean intraoperative pouch capacity, intra-pouch pressure, maximum flow rate, Post void residual volume	Serum creatinine, urine cytology, urine culture, histology	Postoperative histopathology, tumor grading	Recurrence rate
Obara et al., 2006 [14]	Retrospective	31	Maximum functional capacity, max and average flow rate, voiding period, volume of residual urine, pressure of maximum capacity, daytime and nighttime urinary incontinence	Serum urea nitrogen, creatinine, chloride and arterial bicarbonate	Oncological outcomes not mentioned	Survival rates not mentioned
Boonchai et al., 2023 [15]	Retrospective	54 (90)	Maximum cystometric capacity, neobladder compliance, maximum flow rate and post void residual volume	Serum creatinine level and the estimated glomerular filtration rate (eGFR)	Pathological T and N staging, grading,	Survival rates not mentioned
Lantz et al., 2010 [16]	Retrospective	31	Day and nighttime incontinence (day or night-time pad use reported by patients), urinary flow rates (measured by uroflowmetry), and post-void residual.	Renal function assessed by serum creatinine measurements	Pathological T and N staging	Survival rates not mentioned
Nam et al., 2013 [26]	Retrospective	19 (50)	Maximum bladder capacities, maximum filling pressure, maximum flow rate, and maximum urethral closure pressure, day- and night-time incontinence	None	Pathological T and N staging	Survival rates not mentioned
Granberg et al., 2008 [36]	Retrospective	59	Day and nighttime incontinence (day or night-time pad use reported by patients), intermittent self-catheterization	None	Histology, tumor staging, grading, lymph node status, pathology	Time to pelvic recurrence, location of pelvic recurrence and treatment of pelvic recurrence Export to Sheets

This study has several limitations. It is a retrospective analysis with a small sample size, which may limit the generalizability of the findings. The follow-up duration, although sufficient to assess early functional and oncological outcomes, may not capture long-term complications or late functional decline. Additionally, continence outcomes were based on patient-reported pad usage, which may be subject to recall bias. Future prospective studies with larger cohorts and standardized assessment tools are warranted to validate these findings.

## Conclusions

While RARC and neobladder are complex procedures that require specialized surgical expertise, they are technically feasible, safe and can offer hope for patients with bladder cancer to have a more natural and functional alternative to the traditional ileal conduit, allowing patients to engage in normal activities.
